# Supplementary material for: Diarrhea treatment center (DTC) based diarrheal disease surveillance in settlements in the wake of the mass influx of forcibly displaced Myanmar national (FDMN) in Cox’s Bazar, Bangladesh, 2018
Source: PLoS One. 2021 Aug 2;16(8):e0254473. doi: 10.1371/journal.pone.0254473 (PMC8328326; doi:10.1371/journal.pone.0254473)
Supplement: Attachment — Submitted filename: Reviewer Comment_PONE-D-20-28913.docx [file pone.0254473.s001.docx]

**Reviewer’s Comment:**

**Article number:** PONE-D-20-28913

**Title of the Article:**

Diarrhea Treatment Centre (DTC) based diarrheal disease surveillance in settlements in the wake of the mass influx of Forcibly Displaced Myanmar National (FDMN) in Cox’s Bazar, Bangladesh, 2018.

**Abstract:**

- Study objectives should be clearly mentioned.
- Materials and methods section should be more informative. For instance, the section should include information on the study design, study population, sampling technique, study tool and data analysis plan.

**Introduction:**

- Background information is nicely organized
- Justification should be stronger.
- Study objective is not clear. It should be clearly mentioned.

**Materials and methods:**

Study design should come first, before study site and so on.

Why Bangladesh nationals were included as the study population? Including Bangladesh nationals might dilute the study findings.

Line 140-150: Unnecessary information.

**Sample size:**

Why the prevalence of enteric pathogen (0.3) was used for estimating sample size? Is this the main outcome variable?

**Data collection:**

How was the quality of the collected data ensured?

**Statistical Analysis:**

Please clearly state what are the outcome and explanatory variables.

Only descriptive statistics seems unsatisfactory for publishing in a renowned journal. Some sorts of inferential statistics should be performed (linear/logistic regression etc.).

**Ethical consideration:**

Please mention whether informed written consent was obtained or not?

What about anonymity and confidentiality?

**Results:**

In the method section, it is stated that the study performed only descriptive analysis, however, in the result section *“P-value”* has been reported. Please explain how was the *“P-value”* obtained.

Formatting of the Tables should be improved by following those in international journals.

**Discussion:**

Discussion section should be enriched by explaining the study findings using existing literature. For instance, authors stated “This may be due to the increased vulnerability of females to diarrheal illnesses because of their higher compromised immunity or excess exposure to contaminated water and food during household activities.” (Line 312-314). This explanation should be backed up by relevant reference.

Please mention the major strengths of the study.

Adding a “Conclusion” section would be beneficial.
