# Supplementary material for: Diarrhea treatment center (DTC) based diarrheal disease surveillance in settlements in the wake of the mass influx of forcibly displaced Myanmar national (FDMN) in Cox’s Bazar, Bangladesh, 2018
Source: PLoS One. 2021 Aug 2;16(8):e0254473. doi: 10.1371/journal.pone.0254473 (PMC8328326; doi:10.1371/journal.pone.0254473)
Supplement: Attachment — Submitted filename: Response to the reviewers and editorial board (1).docx [file pone.0254473.s002.docx]

8 February 2021

**Editor-in-Chief**

**PLOS ONE**

**San Francisco, California, US**

**Subject:** Revised submission of the manuscript, entitled " Diarrhea Treatment Centre (DTC) based diarrheal disease surveillance in settlements in the wake of the mass influx of Forcibly Displaced Myanmar National (FDMN) in Cox’s Bazar, Bangladesh, 2018".

Dear Sir,

Thank you for providing us the opportunity to submit our manuscript following revision. We greatly appreciate the comments and we have attempted to address them in full. We now submit the revised manuscript, which highlights the changes as well as in track change to the comments. We sincerely hope that this manuscript in now suitable for publication in nutrients journal.

Thank you very much.

Yours sincerely,

Dr. ASG, MBBS, MPH

Emeritus Scientist

Nutrition and Clinical Services Division, icddr,b

Dhaka, Bangladesh.

Email. gfaruque@icddrb.org

**Response to the reviewers and editorial board**

| **Comment(s)** | **Response** |
| --- | --- |
| **Abstract:**   - Study objectives should be clearly mentioned. - Materials and methods section should be more informative. For instance, the section should include information on the study design, study population, sampling technique, study tool and data analysis plan. | Authors express sincere gratitude to the reviewer for sharing valuable comments and suggestions. All authors recognize that comments and suggestions are very generous and important to strengthen the manuscript. The revised objectives now read: *The present study aimed to report the characteristics of the hospitalized patients in the newly deployed DTCs, focusing additionally on water and sanitation practices of the families, infant and young child feeding practices, nutritional status of the under-five children and women of childbearing age, and the associated common bacterial enteric pathogens and rotavirus*.  Please see page 1 in the revised version*.*  The materials and methods section read: *The study was conducted in Ukhiya and Teknaf sub-districts of Cox’s Bazar, Bangladesh, and followed a cross-sectional design. The study population was forcibly displaced Myanmar nationals (FDMN) living in the largely scattered settlements as well as Bangladesh nationals residing in the neighbourhood who sought care at the icddr,b run DTCs, and enrolled into the facility based diarrheal disease surveillance. In total 1792 hospitalized individuals were considered as study participants. Other than relevant data, a single, stool specimen was collected, immediately one step rapid visual diagnostic test for Vibrio cholerae was performed by the Crystal VC dipstick test kit. The provisionally diagnosed specimens of cholera cases were inoculated into Cary-Blair Transport Medium; then sent to the Clinical Microbiology Laboratory of icddr,b in Dhaka, Bangladesh as soon as possible to isolate the colony as well as perform antibiotic susceptibility tests. Data were analysed by STATA (version 15.0 IC, College Station, TX: StataCorp LLC) and analyses included descriptive as well as for analytic methods.*  Please see page 1 and 2 in the revised version. |
|  | |
| **Introduction:**   - Background information is nicely organized - Justification should be stronger. - Study objective is not clear. It should be clearly mentioned. | As per suggestion, necessary additions and revisions have been made. Please see page 3 line 58 in the revised justification section now reads: *During the ongoing threats of cholera outbreaks [26], limited information was available among displaced and host population living in camps and neighbourhood host community on the water and sanitation (WASH) practices of the families, infant and young child feeding (IYCF) practices of children aged 0-35 months, nutritional status of children and women of childbearing age. Moreover, there was a dearth of knowledge about the common associated bacterial enteric pathogens and rotavirus in hospitalized patients in that area. Additionally, it became essential in detecting any disease outbreak immediately, particularly cholera and shigellosis so that early warning and response system can take prompt measures before any spread; thus, the control strategies are less difficult, and more effective in avoiding unexpected deaths.*  Please see page 7 and 8 in the revised version.  The revised study objective now reads: *We aimed to report results from our ongoing diarrheal disease surveillance efforts in Ukhiya and Teknaf sub-districts of Cox’s Bazar, specifically on patients who were hospitalized in the newly deployed icddr,b operated DTCs serving FDMN and the host community populations during April-December, 2018. We would also like to report on WASH practices of the families hospitalized with diarrhoea; IYCF practices of those aged 0-35 months hospitalized in DTCs, nutritional status of the under-five children and women of childbearing age, and the associated common bacterial enteric pathogens and rotavirus.*  Please see page 7 and 8 in the revised version. |
| **Materials and methods:**  Study design should come first, before study site and so on.  Why Bangladesh nationals were included as the study population? Including Bangladesh nationals might dilute the study findings.  Line 140-150: Unnecessary information. | Thank you for spotting this.  *All required revisions have been made now.*  *Now the study design is placed before the study site and so on. Please see page 3 line 59-60 in the revised version.*  *The host population was included in the study because they were living nearby and sought care from the immediate catchment areas of the DTCs, they had easy access to the settlements, their mixing with the displaced population was mostly due to cultural similarities, and they shared equal threats of disease outbreaks. Moreover, from disease epidemiology perspectives, there were possibilities of any rapid spread of disease outbreaks from the neighbourhood host population to the displaced vulnerable population. The study will assist in understanding several differentials when compared between displaced population and host population.*  Please see page 8 and 9 in the revised version.  *Thank you very much for spotting this. All necessary deletions have also been made.* |
| **Sample size:**  Why the prevalence of enteric pathogen (0.3) was used for estimating sample size? Is this the main outcome variable? | Gross revisions have been undertaken as per comments and suggestions. Please see page 3 line 59-60 in the revised version which now reads:  *To calculate sample size, we assumed the proportion of the main outcome variable the females aged 15 years and above hospitalised with acute watery diarrhea episodes which was 63%, desired precision as 3%, and 5% level of significance, the minimum sample size was 995. Considering 5% non-response rate the total sample size of the cross-sectional study design was ~1050.*  Please see page 9 in the revised version. |
|  | |
| **Data collection:**  How was the quality of the collected data ensured? | Thank you for raising this issue We have added a statement under data collection section in page 4 lines 99-100 that reads: *Field research assistants involved in administering field-tested questionnaires were university graduates in any discipline, with minimum of 15 years of schooling. They had an average of 8 years’ work experience in carrying out similar activities. Each morning, the supervisor checked the precision of all anthropometric instruments and calibrated the equipments. Frequent spot checks were conducted to observe data collection process including performance in rapid diagnostic tests. Any detected error was immediately resolved.*  Please see page 10 in the revised version. |
| **Statistical Analysis:**  Please clearly state what are the outcome and explanatory variables.  Only descriptive statistics seems unsatisfactory for publishing in a renowned journal. Some sorts of inferential statistics should be performed (linear/logistic regression etc.). | Authors thank to reviewer for important insight. The paragraph has been revised to incorporate analysis details. We agree with the reviewer’s concern. Please see the revised version page 6 lines 143-149 now reads: *Study population (displaced and host population) was our outcome variable of interest. Explanatory variables included in the analysis were; demographic characteristics: age and sex; clinical features: duration of diarrhea, stool character, dehydration status, and ORS use; nutritional status: type of child nutrition, severe malnutrition of children, breastfeeding status of children 0-23 months old, and nutritional status of women 15-49 years; environmental factors: water source, and type of toilet use pattern; and associated common enteric pathogens: Vibrio cholerae* O1*, Shigella, Salmonella, and rotavirus.*  *Data were analysed by STATA (version 15.0 IC, College Station, TX: StataCorp LLC) and analyses included descriptive as well as for analytic methods. Descriptive statistics were used to summarize the data, including frequencies and proportions for categorical variables. Characteristics based on the exposure were compared using Pearson X^2^ tests for categorical variables. Several statistical plots such as histograms, bar diagrams, pie charts and scatterplots were used for data visualization.*  *Categorical explanatory variables were coded as: age of the female respondent (15 years and more=1, <15 years=0), sex (female=1, male=0), duration of diarrhea (≥1 day=1, <1 day=0); dehydration status (some-severe=1; none=0); ORS use at home (no=1, yes=0); drinking water source (public tap=1, other=0); user of pit latrine without water seal (yes=1, other=0); stool character (watery=1, other=0); and rotavirus (yes=1, no=0).*  *All explanatory variables were analysed initially in a univariate model, and the attributes that were observed to be significantly associated (p-value <0.05) with the outcome variable (study population) were considered to be included in the multivariable logistic regression model.*  *Finally, logistic regression was used to assess the relationship between the outcome variable and explanatory variables with a binary outcome. First, simple logistic regression analyses were undertaken for both outcome and explanatory variables to examine the association between the outcome variable and all explanatory variables separately. Variables that were observed to be significantly associated (p-value <0.25) with the outcome variable (study population) along with clinically relevant non-significant but important variables that have public health implications were included in the multivariable logistic model using backward stepwise selection method. A Goodness of fit test was run to test whether the model fitted well or not. Odds ratios (ORs) with 95% confidence intervals (95% CIs) were estimated to assess the strength of association between outcome variable and the explanatory variables of interest, and p-value<0.05 was considered as the level of significance. We also checked multicollinearity among explanatory variables using variance inflation factor (VIF). No variable with a VIF> 5 was identified.*  Please see page 10, 11 and 12 in the revised version. |
|  |  |
| **Ethical consideration:**  Please mention whether informed written consent was obtained or not?  What about anonymity and confidentiality? | We have revised the paragraph on ethical consideration which now reads: *The present study entitled: “Surveillance for etiologic agents, care-seeking behavior, the status of IYCF and WASH practices among patients or their caregivers from Rohingya refugees as well as host population in Cox’s Bazar district attending icddr,b operated Diarrhea Treatment Centers” was approved by icddr,b’s institutional review board (consists of Research Review Committee and Ethical Review Committee). The data collection process was understood to cause no harm including psychological distress to the participants. Data collection, measurements of nutritional status and collection of stool specimen were carried out after obtaining informed written consent from the respondents. In case of children, the consent of their parents and/or guardians were obtained. If they were unable to read, the consent form was read aloud to the participants or their guardians/parents. A copy of the consent form was given to the respondents for their reference. The consent form was written in simple Bangla language, so that it is easily understood by the participants, even those with little or no formal schooling. Participants who were 11-17 years of age, in addition to their assent, consent of their parents and/or guardians were also obtained. The enumerators made it clear to the participant that answering the questions will not cause any risk to him/her or his/her family, and there would be no direct benefit to him/her or his/her child responding to the questionnaire. Moreover, their participation in the study might serve as a groundwork for an intervention programme among the displaced and host population that would try to benefit him/her and others by delivering improved health care services. Participants were informed that no money would be provided to them or their family members for participation, and they would not have to pay any remuneration for participating in the study. They were also informed about their rights to withdraw themselves at any point of the interview as well as from the study. The precaution was taken to keep information about the participants confidential. Identity of the participants was stored in locked cabinets and in password-protected computer files and was available to the key researchers only.*  *The dataset contained name and address of the participants, but those were not used during the analysis, writing of the report or the manuscript.*  Please see page 12 in the revised version. |
| **Results:**  In the method section, it is stated that the study performed only descriptive analysis, however, in the result section *“P-value”* has been reported. Please explain how was the *“P-value”* obtained.  Formatting of the Tables should be improved by following those in international journals. | Thank you for spotting this error. Please see the revised version which reads: *Variables with a p<0.05 in the final regression model was considered as the level of significance.*  Please see page 16-19 in the revised version. |
| **Discussion:**  Discussion section should be enriched by explaining the study findings using existing literature. For instance, authors stated “This may be due to the increased vulnerability of females to diarrheal illnesses because of their higher compromised immunity or excess exposure to contaminated water and food during household activities.” (Line 312-314). This explanation should be backed up by relevant reference. | Vulnerability of pregnant and lactating women and school-aged girls to increased rates of diarrheal diseases including cholera has been repeatedly indicated by several studies. Researchers have postulated that severely malnourished women are likely to have compromised immune systems due to diminished nutritional supply that often fails to meet their needs in emergency and crisis settings, thus they are becoming more prone to diarrheal diseases including cholera. Women and school going girls are more exposed to higher infective doses of causative agents because of their active involvement in domestic activities such as taking care of sick family members, washing their dirty clothes, and cleaning toilets. Additionally, they are involved in fetching water, handling untreated water and raw food particularly during cooking which could ultimately become contaminated. Thus, their existing immunity is at risk to be superseded by the infective dose of an enhanced number of causative organisms [31–36].  Please see page 20 in the revised version. |
| Please mention the major strengths of the study. | The major strengths of the study now read: *A large number of patients and quality laboratory performance were the strengths of the study. The study has highlighted the importance of DTC-based diarrheal disease surveillance with round-the-clock capturing of patients with a timely laboratory back-up and immediate reporting which can enhance the preparedness and response team in early detection of outbreaks followed by the institution of preventive and control measures in emergency and crisis settings. Moreover, DTC based surveillance facilitated direct comparisons between the displaced population and the host community population concerning a good number of key variables.*  Please see page 26 in the revised version. |
| Adding a “Conclusion” section would be beneficial. | We agree with the reviewer’s suggestion. The statement related to conclusion stands: *With effective DTC networking, laboratory- based diseased surveillance, and OCV campaigns in the present emergency and crisis settings, no cholera or acute watery diarrhea outbreak was observed during the study period. Preparedness for surges is warranted in this emergency and crisis settings.*  Please see page 26 in the revised version. |
