# Supplementary material for: Diarrhea treatment center (DTC) based diarrheal disease surveillance in settlements in the wake of the mass influx of forcibly displaced Myanmar national (FDMN) in Cox’s Bazar, Bangladesh, 2018
Source: PLoS One. 2021 Aug 2;16(8):e0254473. doi: 10.1371/journal.pone.0254473 (PMC8328326; doi:10.1371/journal.pone.0254473)
Supplement: Attachment — Submitted filename: Response to reviewers comment.docx [file pone.0254473.s003.docx]

Date: 30 May, 2021

To: Editor

PLOS One

From: Dr. ASG Faruque

Emeritus Scientist

Nutrition and Clinical Services Division, icddr,b

**Subject:** Response on the comments of the reviewers of PLOS One

Article number: PONE-D-20-28913-R1 entitled **“Diarrhea Treatment Centre (DTC) based diarrheal disease surveillance in settlements in the wake of the mass influx of Forcibly Displaced Myanmar National (FDMN) in Cox’s Bazar, Bangladesh, 2018”**

Dear Sir,

Thank you for providing us the opportunity to resubmit our manuscript following revision. We greatly appreciate helpful and precise comments from the respected reviewers and we have attempted to address them in full. We now submit the revised manuscript, which highlights the changes in track change, and a fresh revised version, as well as this cover letter which provides a point-by-point response to the reviewers’ comments.

We sincerely hope that this manuscript is now suitable for publication in your prestigious journal: PLOS one

Thank you.

**Reviewer #1: Reviewer’s Comment:**

Article number: PONE-D-20-28913-R1

Title of the Article:

Diarrhea Treatment Centre (DTC) based diarrheal disease surveillance in settlements in the wake of the mass influx of Forcibly Displaced Myanmar National (FDMN) in Cox’s Bazar, Bangladesh, 2018.

Overall:

The manuscript reported some important and interesting findings and used data from a satisfactorily designed study. However, the way the manuscript was written mostly seems with a report and not a journal article. A journal article usually identifies 2-4 specific research questions and keep focus to answer those questions throughout the document. In case of the current manuscript, that consistency and coherence is not properly maintained and the study objective is too broad. I would, therefore, suggest the authors to kindly identify 1-2 important research questions (e.g., nutritional status of women and children and their associated factors, WASH practice and its associated factors etc.) and keep focus on those aspects in the revised manuscript.

**Response:** Thank you very much for your valuable comment. As the threats of diarrheal disease outbreaks including cholera and shigellosis were prevailing in the settlements, therefore for better preparedness and mitigation of threats a network of DTCs was established mainly to serve the displaced population living in the settlements. Given the fact that hospitalization of the displaced population from the settlements was lower than that of host population, therefore we decided to address the research question of such a relatively low care seeking practices and assist in formulation of improved health services strategy by determining factors that were associated with hospitalization in DTCs of FDMN from settlements.

Table 6. Factors associated with hospitalization of displaced population from settlements in DTCs, April - December 2018, Cox’s Bazar, Bangladesh

**Abstract:**

In the abstract, it is mentioned that “In total 1792 hospitalized individuals were considered as study participants”, however, in the main manuscript, sample size was estimated as 1050 (L 174). Kindly explain the discrepancy.

**Response:** Thank you very much for your kind observation and asking us to explain the discrepancy. We have revised that section to explain the discrepancy as much as possible by us. Thus, to calculate sample size, we assumed the proportion of the main outcome variable the displaced population hospitalized with acute watery diarrhea episodes as 40%, desired precision as 2.5%, 5% level of significance, the minimum sample size was 1475. Considering a 10% non-response rate the minimum sample size for this cross-sectional study design was 1639. The study had an analyzable sample size of 1792 hospitalized individuals. Line 168-172

**Study objective:**

A journal article usually identifies 2-3 specific study objectives and keep focus to those throughout the manuscript. In case of the current manuscript, the study objective is too broad (to report the characteristics of the hospitalized patients in the newly deployed DTCs, focusing additionally on water and sanitation practices of the families, infant and young child feeding practices, nutritional status of the under-five children and women of childbearing age, and the associated common bacterial enteric pathogens and rotavirus). Moreover, consistency and coherence among study objective, method (specially outcome explanatory variables) and result is not properly maintained. I would, therefore, suggest the authors to kindly identify important key topics (e.g., nutritional status of women and children and their associated factors, WASH practice and its associated factors etc.) and keep focus on those aspects in the revised manuscript.

**Response:** Thank you for your kind suggestions. We have made necessary revisions by adding a few sentences to the text of the manuscript. To maintain consistency and coherence among study objective, method, and result we aimed to report results from our ongoing diarrheal disease surveillance efforts in Ukhiya and Teknaf sub-districts of Cox’s Bazar, specifically on patients who were hospitalized in the newly deployed icddr,b operated DTCs serving FDMN and the host community populations during April-December, 2018. We would also like to report on WASH practices of the families hospitalized with diarrhea; IYCF practices of those aged 0-35 months hospitalized in DTCs, nutritional status of the under-five children and women of childbearing age, and the associated common bacterial enteric pathogens and rotavirus. Additionally, we aimed to highlight the factors that were associated with the hospitalization of displaced population in the DTCs. Line 135-141.

**Materials and methods**

**Sample size:**

Authors considered the females aged 15 years and above hospitalized with acute watery diarrhea as the main outcome variable for sample size calculation (L 170-171), however, in the Statistical Analysis section (L 2014), they stated “Study population (displaced and host population) was our outcome variable”. Please explain this discrepancy.

**Response:** Thank you very much for your valuable suggestion. To calculate sample size, we assumed the proportion of the main outcome variable displaced population hospitalized with acute watery diarrhea episodes as 40%, desired precision as 2.5%, 5% level of significance, the minimum sample size was 1475. Considering a 10% non-response rate the minimum sample size for this cross-sectional study design was 1639. The study had an analyzable sample size of 1792 hospitalized individuals. Line 168-172.

**Statistical Analysis**

L 204-210: Authors stated “Study population (displaced and host population) was our outcome variable of interest. Explanatory variables included in the analysis were; demographic characteristics: age and sex; clinical features: duration of diarrhea, stool character, dehydration status, and ORS use; nutritional status: type of child nutrition, severe malnutrition of children, breastfeeding status of children 0-23 months old, and nutritional status of women 15-49 years; environmental factors: water source, and type of toilet use pattern; and associated common enteric pathogens: Vibrio cholerae O1, Shigella, Salmonella, and rotavirus”. This means in this study, the displaced and host population (outcome variable) was explained by (or depends on) age, sex, nutritional status, water source etc., which is logically not possible. Most importantly, the outcome variable should be any characteristic of study population and study population itself cannot be an outcome variable. Authors are kindly requested to clearly think about the outcome variable and explanatory variables and then perform analysis accordingly.

**Response:** Thanking once again for the valuable observations. Necessary revisions have been made in the manuscript accordingly. In this study, the differential characteristics of the hospitalized displaced and host population (outcome variable) were explained by age, sex, nutritional status, water source etc. The outcome variable was hospitalization of study population. Line206-207.

L 220: Authors stated that "All explanatory variables were analyzed initially in a univariate model, and the attributes that were observed to be significantly associated (p-value <0.05) with the outcome variable (study population) were considered to be included in the multivariable logistic regression model". Why did the authors perform univariate analysis for explanatory variables but not for outcome variable given that for further analysis, univariate analysis of outcome variable is also important?

**Response:** Our sincere thanks for sharing your kind opinion. We performed univariate analysis for outcome variable. Those texts that were duplicated were removed and we made necessary revisions. Line 217-218.

L 230: Authors mentioned “A Goodness of fit test was run to test whether the model fitted well or not”. Which Goodness of fit test was used?

**Response:** Thanks a lot for your kind suggestion and asking us to address your query. We have responded by adding the name of the Goodness of fit test. The Hosmer–Lemeshow test, a statistical test for goodness of fit was used for logistic regression models in this analysis. Line 229-230.

L 233: The statement “p-value<0.05 was considered as the level of significance” is not correct. Level of significance (expressed as alpha or α) is the probability of rejecting the null hypothesis when it is true, whereas, p-value is the probability that an observed difference could have occurred just by random chance. P-value and α are inter-related, however, a p-value cannot be considered as the level of significance.

**Response:** Many thanks for sharing your concerns. We have made necessary revisions in the text accordingly. A p-value <0.05 indicated strong evidence against the null hypothesis. Line 230.

**Results**

L 316: The finding presented in the Table 3 indicates Nutritional status of children is the outcome variable and its distribution is shown across the type of population (all, displaced and host), which does not match with the outcome variable stated in the statistical analysis section (L 204). Please clearly identify the outcome variable of this paper.

**Response:** Thanks once again for your kind suggestions. The outcome variable was the hospitalized study population. Line 206-207.

L 356: The sub-heading “Factors associated with hospitalization of the displaced population in DTCs” suggests that authors intended to identify which factors affected hospitalization of the displaced population, however, Table 6 indicates that they tried to compare WASH indicators (and NOT factors of hospitalization) between displaced and host population. On the other hand, the description under the sub-heading (e.g., hospitalized FDMN were significantly less likely to pass watery stool etc.) implies that the authors intended to identify difference in WASH indicators (such as watery vs. bloody diarrhoea) among hospitalized FDMN. Authors are kindly requested to thoroughly revise Table 6 and related description in the text to ensure alignment. Also, for multivariate regression tables, please report categories of covariates including reference category (e.g., variable: gender; category: male vs female, reference category: male). Please follow other articles published in Plos One for table formatting.

**Response:** While thanking for your kind valuable suggestions we would like to mention that accordingly necessary revisions have been made in the manuscript as well as in Tables of the manuscript. The association of explanatory variables after adjusting for covariates with outcome variable revealed that hospitalized FDMN were significantly more likely to report after 1 day and more (aOR 1.15, 95% CI 1.01, 1.31), drinking water from public tap (aOR 17.82, 95% CI 12.17, 26.10), user of pit latrines without water seal (aOR 4.06, 95% CI 3.10, 5.31), not user of ORS at home before coming to the DTCs (aOR 1.89, 95% CI 1.49, 2.39), and less likely to get admission with rotavirus diarrhea (aOR 0.59, 95% CI 0.46, 0.75) and watery stool (aOR 0.76, 95% CI 0.59, 0.98) (Table 6). Necessary revisions in Table 6 have been made. Line 376-378.
